# Lite-3DCNN Combined with Attention Mechanism for Complex Human Movement Recognition

**DOI:** 10.1155/2022/4816549

**Published:** 2022-09-09

**Authors:** Maochang Zhu, Sheng Bin, Gengxin Sun

**Affiliations:** College of Computer Science & Technology, Qingdao University, Qingdao 266071, China

## Abstract

Three-dimensional convolutional network (3DCNN) is an essential field of motion recognition research. The research work of this paper optimizes the traditional three-dimensional convolution network, introduces the self-attention mechanism, and proposes a new network model to analyze and process complex human motion videos. In this study, the average frame skipping sampling and scaling and the one-hot encoding are used for data pre-processing to retain more features in the limited data. The experimental results show that this paper innovatively designs a lightweight three-dimensional convolutional network combined with an attention mechanism framework, and the number of parameters of the model is reduced by more than 90% to only about 1.7 million. This study compared the performance of different models in different classifications and found that the model proposed in this study performed well in complex human motion video classification. Its recognition rate increased by 1%–8% compared with the C3D model.

## 1. Introduction

In recent years, with the rapid development of deep learning, computer vision has made rapid progress, and human action recognition has become a research field that has attracted much attention. Despite the continuous improvement of research in this field, there are still many challenges for complex human action recognition in videos.

A 3D convolution network (3DCNN) [1] is widely used in human motion recognition. It is improved based on 2D-CNN [2] and modeling time information through 3D convolution and 3D pooling operation to extract spatio-temporal details in videos. However, the video of complex human movement has complex semantics and a lot of redundant information, such as background clutter, occlusion, and high dimensional data, which bring a lot of difficulties to motion recognition. At the same time, the existing neural network based on 3D convolution has a colossal structure, which requires a lot of computing space and time due to its high requirements on hardware devices. Due to their complex network structures, these deep learning models are incompatible with devices with limited computing and storage space, such as smartphones, tablets, and PCS. Therefore, designing an efficient and lightweight motion recognition algorithm is very important.

In this study, the traditional three-dimensional convolutional neural network framework is improved to reduce the number of convolution kernels and the number of convolution operations. Meanwhile, 3 × 3 × 3 pooling kernels are used for pooling operations, and all zero filling is not used. The self-attention mechanism is added in the final feature extraction stage to establish the connection between spatial pixels. Finally, the softmax layer is used to classify complex human movements. Experimental results show the effectiveness of the proposed algorithm. This method does not use complicated and computationally expensive networks, such as C3D-bidirectional LSTM Net [3] or PWCNet [4], to extract time features from test videos. Instead, the simplified C3D Net was used to extract spatial and temporal features through adequate data pre-processing and then integrated with the attention mechanism [5] to extract global features as much as possible with limited parameters and computation. The trainable parameters of the Lite-3DCNN network structure proposed in this study are reduced to about one-thirtieth of the original C3D network. The main contributions of this work are as follows:

1. Simplify the C3D network structure and propose a lightweight 3DCNN architecture for complex human motion classifiers.
2. The self-attention mechanism is integrated to enhance the learning of dependent features and global features of video frame sequences.
3. Compared with the traditional C3D network, the recognition accuracy of this method is slightly improved, and the number of parameters is significantly reduced.

## 2. Related Work

In recent years, human motion recognition based on video has become one of the most popular research fields in computer vision and pattern recognition [6]. It has various applications, such as surveillance, robotics, healthcare, video search, and human-computer interaction. Human motion recognition in the video involves many challenges, such as cluttered backgrounds, occlusion, viewpoint changes, execution rate, and camera movement. Over the decades, several technologies have been proposed to address these challenges.

The framework for action recognition can be divided into two types. One is to create a single network and combine two-dimensional CNN with an RNN. In literature [7], the author first uses a convolutional neural network to extract spatial features. The convolutional layer is followed by RNN (recursive recurrent neural network), which allows time information to flow in time steps. Then, time pooling is used to aggregate the features of all time steps to form video sequence features.

The other is the framework based on 3DCNN [8], which uses three-dimensional convolution to extract spatial features. For example, in reference [9], the author extended the convolutional neural network to 3D to automatically learn spatio-temporal features. Then, a recurrent neural network is trained to classify each sequence considering the time evolution of each time step's learning features. The authors of [10] proposed a method of deep learning to recognize human actions based on motion sequence information in RGB-D video. A new representation emphasizes the critical postures associated with each step. Features obtained from motion in RGB and deep video streams are input to the convolutional neural network to learn distinguishing features. Similarly, Wang and Dantcheva [11] trained and fine-tuned 3D ResNet [12] on the well-known FaceForensic++ dataset, which is an excellent motion recognition network [13]. In addition, generative adversarial networks (GAEL Net [14]) have also been used to design robust facial manipulation detectors. Therefore, researchers began designing more complex architectures to achieve higher detection accuracy. A method of combining 3DCNN with ConvLSTM was proposed in [15] and applied to human action recognition. The 3DCNN model proposed in [16] addresses a complex scene classification problem. It uses the spatial and temporal features of the video to classify scenes as helping or non-helping in natural disasters. The authors of [11] proposed to use exponential linear unit-3D convolutional neural networks to extract deep features of moving videos to represent videos. The ability of state-of-the-art video CNNs (including 3D ResNet, 3D ResNet, and I3D) to detect tampered videos is investigated in work [17]. The authors of [18] proposed a method for anomaly detection in crowd scenes. They offered a 3DCNN architecture and a 3D GAN for domain adaptation to reduce the domain gap. The authors of [19] proposed a method to extract kinematic pose features from 3D joint positions. It is used to classify Support Vector Machines (SVM) and Convolutional Recurrent Neural Networks (CRNN). Vehicle behavior recognition is performed using 3DCNN in the article [20].

These high-precision motion classifiers have huge network scale and complexity. When experiments are carried out on the Utd-MHAD dataset, both the decision level and feature level fusion methods produce higher identification accuracy than those using each sensor mode alone. The highest accuracy of the decision level fusion method [21] is 95.6%. However, it consists of about 27 M trainable parameters. Similarly, networks based on pre-trained VGG-16 [22], ResNet [23], 3D ResNet [12], and optical flow-based methods [24] are networks with high computational costs. Due to their large size and computing power, these efficient networks are incompatible with limited computing and space devices such as smartphones, personal laptops, and tablets. However, lightweight deep learning models are easier to train and less expensive to update when deployed on smartphones, personal laptops, and tablets.

Considering many real-life application scenarios, the deep learning action classification model has been widely used in PCs and personal laptops. Laptop computers carry out many human motion recognition scenarios, and the current configuration of laptop computers is often unable to achieve training and use a large structure of deep learning models. As a result, real-world applications place high demands on lightweight models. Therefore, this paper proposes a light 3D convolutional neural network (Lite-3DCNN) for complex human motion classification.

## 3. Proposed Method

**Figure 1:**
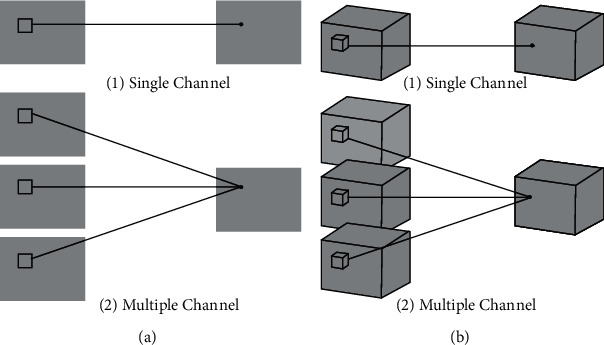
2D Convolution (a) and 3D convolution (b) diagram.

The 3D convolutional network is an extension based on the 2D convolution, which adds the time dimension to the 2D convolution to fully use the timing information in the video, as shown in Figure 1. It is widely used in video classification and retrieval.

However, the traditional 3D convolutional network framework has huge parameters and requires high machine performance. These models' training and prediction stages consume a lot of time and computing power. At the same time, sports videos often contain high-level semantic information and a large amount of redundant data, and videos of different modes interfere with each other, making the model unable to accurately capture essential features in complex videos. This study optimizes based on the C3D framework, first reducing the number of convolution operations and increasing the size of the pooling kernel, which significantly reduces the computational complexity. Then, a PreLU activation function with learnable parameters is used to avoid the problem of vanishing gradients. A self-attention mechanism is introduced into the model to extract long-distance interdependent features in complex videos, dramatically enhancing the feature extraction capability of lightweight 3D convolutional networks. In this study, data pre-processing is performed using average frame skip sampling and scaling. One-hot encoding is performed on the data, which enriches the training data features and makes the calculation between the elements more reasonable. The experimental results in Section 4 demonstrate that the method proposed in this study is suitable for complex human motion classification and is an efficient model that is convenient for training and prediction.

**Figure 2:**
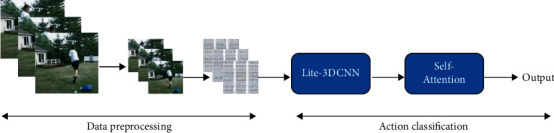
The overall process of this method.

The proposed approach is divided into two phases, as shown in Figure 2. The first stage is the pre-processing stage. The video is clipped and scaled to continuous video frames, and then the four-dimensional matrix with depth is transformed. The four-dimensional matrix comprises the video frame's length, width, channel number, and depth. The transformed four-dimensional matrix plus batch_size is used as the input of the 3D convolution operation in the classification stage. In the second stage, the five-dimensional matrix calculated in the pre-processing stage is used as the input of Lite-3DCNN in the detection stage. The input five-dimensional matrix consists of the batch size, the width, and height of the video frame, and the depth and channel number, respectively, i.e., [batch_size, width, height, depth, channel]. Lite-3DCNN processes the input and learns the key features. Finally, an autonomous attention mechanism is added to enhance the learning of long-term dependent features.

### 3.1. Data Preprocessing

**Figure 3:**
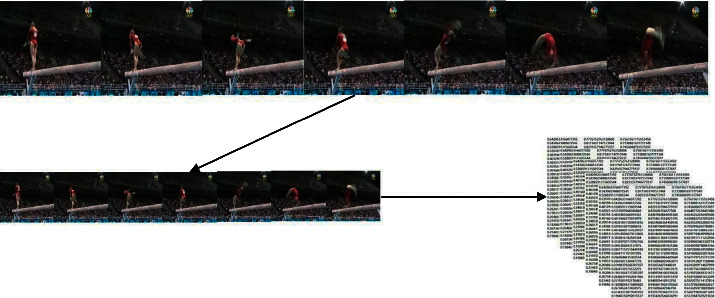
Data pre-processing.

First, OpenCV was used to clip the video 20mmc20 times. Since the video data length is inconsistent with intercepting the image samples as evenly as possible, the clipping method uses average skipping frame sampling. The depth of a video frame is the number of times a video is clipped. *frames*_*total*_ represents the total number of frames for a video, *de* *pth* indicates the number of frames you want to intercept, and *output*_*frames*_ means the video frame set after clipping, as shown in formula (1) and Figure 3,

(1) $$\begin{array}{c} output_{frames}=\overset{depth}{\underset{i=0}{\sum }}\frac{i*frames_{total}}{depth}\text{.} \end{array}$$

Then, resize it to 32 × 32 and store all the processed images, including the video frame's width and height and height of the video frame and the number of channels. Finally, all the videos in each category are traversed, and then the four-dimensional array obtained after each video processing is combined to form a five-dimensional *X*.

**Figure 4:**
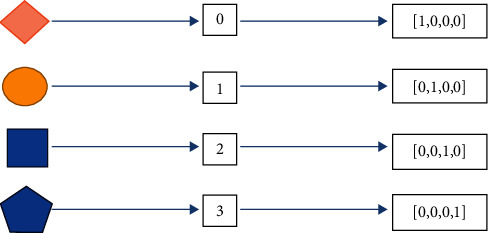
One-hot encoding.

As the input_shape format of Conv3d required, the data dimensions were adjusted to suitable inputs using the transpose method. Finally, the input data is *X*, the label is *Y*, and the label *Y* is processed by one-hot encoding [25], which makes the feature calculation among features more reasonable and improves the computing speed. The calculation method is shown in Figure 4.

### 3.2. Action Classification Model

**Figure 5:**
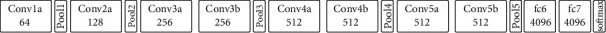
C3D network structure.

The original C3D [8] network consists of five pool layers, eight 3D convolutional layers, and then two fully connected layers; the last one is the softmax layer for action prediction, in which the number of convolution cores in each convolution layer is 64, 128, 256, 256, 512, and 512. All pooling layers are maximum pooling, with the first pooling layer having a kernel size of 1 × 2 x 2 (in order not to merge time signals prematurely). The size of the remaining convolution kernel is 2 × 2 x 2, and the maximum pooling step is 1, which means that the size of the output signal is reduced by eight times compared with the input signal. The C3D network structure is shown in Figure 5.

The Lite-3DCNN network structure proposed in this study contains only four convolution operations, and the size of the convolution kernel at each layer is reduced to 32, 32, 64, and 64. At the same time, the ReLU activation function in the original network was abandoned in this paper. PreLU and softmax activation functions were used alternately after each convolution layer for activation operation.

**Figure 6:**
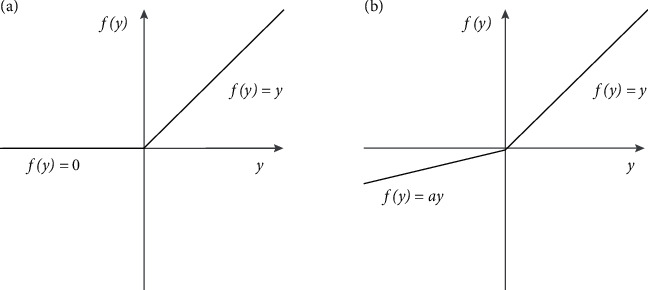
Activation function comparison. (a) ReLU (*x*) and (b) PReLU (*x*).

According to Figure 6 and formula (2), the gradient of the ReLU activation function is 0 when *x* < 0. Hence, the rise of this neuron and subsequent neurons is always zero, which is gradient disappearance. In formula (2), in PReLU, the slope *a*_*i*_ of the negative part is not defined in advance but is constantly updated through backpropagation, as shown in formula (3). In this way, the problem of gradient disappearance can be solved, and the classification accuracy can be improved only by adding a few parameters,

(2) $$\begin{array}{c} \left\{\begin{array}{c} ReLU\left(x\right)=\left\{\begin{array}{c} x,ifx<0\\ 0,ifx\geq 0 \end{array}\right.\\ PReLU\left(x\right)=\left\{\begin{array}{c} x_{i},ifx_{i}<0\\ a_{i}x_{i},ifx_{i}\geq 0 \end{array}\right. \end{array}\right.\text{,} \end{array}$$

(3) $$\begin{array}{c} \Delta a_{i}=U\Delta a_{i}+\epsilon \frac{\partial y}{\partial a_{i}}\text{,} \end{array}$$

where **U** represents the momentum, *ε* represents the learning rate, and the initial **a**_**i**_ is 0.25. Experiments show that the PreLU function can accelerate model convergence and improve classification accuracy.

**Figure 7:**
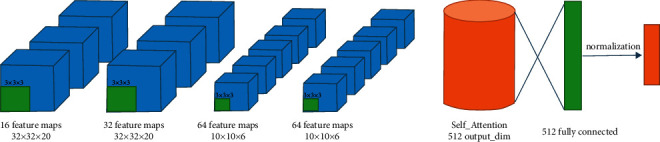
Action recognition architecture of this study.

The proposed method only uses maximum pooling twice, changing the size of the pooling kernel to 3 × 3 × 3 and further reducing the number of parameters on the premise of sacrificing a few features. To extract the most valuable elements from the limited number of features, the self-attention mechanism with 512 output dimensions was connected to the full connection layer with the same number of units before the softmax classification layer at the end of the model, and then normalized and finally sent to the output layer. The pre-processed data is input into the Lite-3DCNN network, and high-level semantic information is collected at a higher level of the deep convolutional network. Next, the Self_Attention network identifies long-term motion correlations from features extracted by 3D convolution. Therefore, the Lite-3DCNN coupled Self_Attention architecture proposed by us can better extract the spatio-temporal features of data while minimizing the time and space complexity. The complete network architecture information is shown in Figure 7.

**Figure 8:**
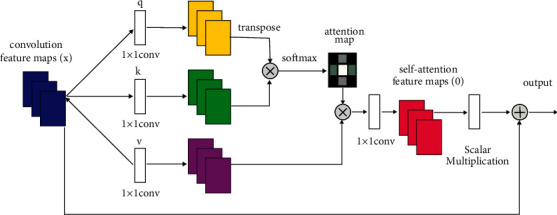
Self-attention mechanism unit.

Self-attention is borrowed from natural language processing, so it retains names like query, key, and value. The input convolution feature maps are feature maps extracted by backbone CNN. The structure of self-attention is divided into three branches from top to bottom: query key and value.  Figure 8 is the basic structure of self-attention, and the calculation formula is shown in formula (5),

(4) $$\begin{array}{c} Attention\left(Q,K,V\right)=softmax\left(\frac{QK^{T}}{\sqrt{d_{k}}}\right)V\text{,} \end{array}$$

where *Attention*(*Q*, *K*, *V*) refers to the value of attention obtained and *Q*, *K*, and *V* are the query vector matrix, key vector matrix, and value vector matrix, respectively. Each row in these three matrices represents a corresponding vector. *Q*, *K*, and *V* are typically obtained by multiplying the input sequence *X* by three matrices, *W*^*q*^, *W*^*k*^, *W*^*v*^.

First, for each sample, we have a *d*_*q*_-dimensional query vector, forming an *N* × *d*_*q*_*n*-dimensional query vector matrix *Q*. You can think of the query vector as the characteristic of the model.

Then, for each piece of information (vector) in our “information base,” there is a *d*_*q*_-dimensional key vector and a *d*_**v**_-dimensional value vector, forming a key-value pair. Suppose there are *n*_*v*_ pieces of information, then they constitute the key vector matrix *K* of *n*_*v*_ by *d*_*v*_ dimension and the value vector-matrix *V* of *n*_*v*_ by *d*_*v*_ dimension, respectively. You can think of key vectors as features of information and value vectors as the information content.

QK_n*∗ ***n**_**v**__^T^ represents the similarity between *n* query vectors (sample features) and *n*_*v*_ key vectors (information features). For example, if we assume *n* = 2 and *n*_*v*_ = 3, then the first behavior [2, 3, 5] represents that the similarity between the first sample and the first, second, and third information is 2, 5, and 3 respectively.

We then apply an activation function *ω* (·), typically softmax (·/), to obtain the correlation or similarity distribution *ω* (QK_n*∗ ***n**_**v**__^T^) between samples and pieces of information. For the previous example, we simply call *ω* (*x*) = [*x*_*i*_/∑*x*_*i*_]. The result is [0.2, 0.5, 0.3], which means that the correlation or similarity between the first sample and the first, second, and third information is 20%, 50%, and 30%, respectively.

Finally, multiply *ω* (QK_n*∗ ***n**_**v**__^T^) and *V*_**n**_**v**_*∗* **d**_**v**__, and get *Attention*(*Q*, *K*, *V*), that is, the weighted sum of the value vector (information); the weight is the distribution of correlation or similarity between each sample and each piece of information, and this is the final result of self-attention. The network structure and parameters of this framework are shown in below Table 1.

## 4. Experimental Discussion

### 4.1. The Dataset

**Figure 9:**
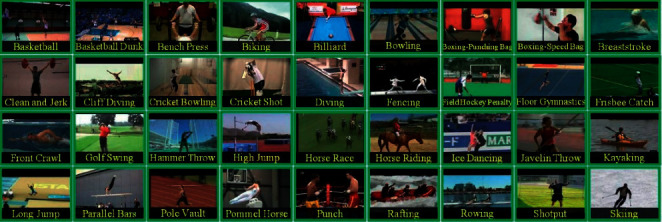
Sports video dataset.

The method was trained and tested on the UCF-101 dataset [24]. The dataset contains videos of different types of sports, such as handstand walking, canoeing, horse racing, etc. The UCF-101 dataset was generated from a collection of YouTube videos, with videos in 101 action categories divided into 25 groups, each of which can be composed of 4–7 action videos. Videos from the same group may have some standard features, such as similar backgrounds, similar viewpoints, etc. They are shown in Figure 9.

At the same time, it offers the most incredible variety in motion, with wide variations in camera movement, object appearance and posture, object proportions, viewpoint, cluttered backgrounds, lighting conditions, and so on, making it the most challenging dataset to date. The original dataset contains 13320 original videos and 50 related sports videos, of which 30 sports videos are randomly selected in this study.

### 4.2. Contrast Experiment

According to the nature of the deep learning model in this study, the video is first processed as video frames. To minimize clipping and retain relatively complete video features, the average structure hopping sampling method is adopted in the data pre-processing stage, and then the video frames are scaled. This paper extracts 20 RGB video frames from each video clip. Each video clip is fed individually into a Lite-3DCNN network stream with a frame size of 20 × 32 × 32.

In the experiment in this paper, the initial learning rate of model training was set at 0.001, the PreLU activation function was used to accelerate model convergence, and the adaptive moment estimation (Adam) optimizer [26] was used during training, which combined the advantages of AdaGrad and RMSProp optimization algorithms. The update step size is calculated using the first moment estimation and second moment estimation.

In formula (5), *β*_1_ is the exponential decay rate, controlling the weight distribution (momentum and current gradient), and *β*_2_ is the exponential decay rate, maintaining the influence of the previous gradient square. *t* is a time step, initialized to 0. *g*_*t*_ is the gradient when the time step is *t*. *θ* is the parameter to be updated, and *f*(*θ*) is the random objective function of parameters. *m*_*t*_ is the first-order moment estimation of the gradient, and *u*_*t*_ is the second-moment estimation of the slope. $\overset{ˇ}{m_{t}}$, $\overset{ˇ}{u_{t}}$ is the correction of $\overset{ˇ}{m_{t}}$ and $\overset{ˇ}{u_{t}}$, respectively. *σ* is the learning rate, and *ε* is a constant to maintain numerical stability.

The specific update rule is as shown in formula (5): initialize *β*_1_ = 0.9, *β*_2_ = 0.999, *ϵ* = 10e − 8, and *σ* = 0.001. The minimum batch of training is 32 samples for data training,

(5) $$\begin{array}{c} \left\{\begin{array}{c} t=t+1\\ g_{t}=\nabla_{\theta }f_{t}\left(\theta_{t-1}\right)\\ m_{t}=\beta_{1}m_{t-1}+\left(1-\beta_{1}\right)g_{t}\\ u_{t}=\beta_{2}u_{t-1}+\left(1-\beta_{2}\right)g_{t}^{2}\\ \overset{ˇ}{m_{t}}=\frac{m_{t}}{1-\beta_{1}^{t}}\\ \overset{ˇ}{u_{t}}=\frac{u_{t}}{1-\beta_{2}^{t}}\\ \theta_{t+1}=\theta_{t}-\frac{\sigma }{\sqrt{\overset{ˇ}{u_{t}}}+\epsilon }\overset{ˇ}{m_{t}} \end{array}\right.\text{.} \end{array}$$

**Figure 10:**
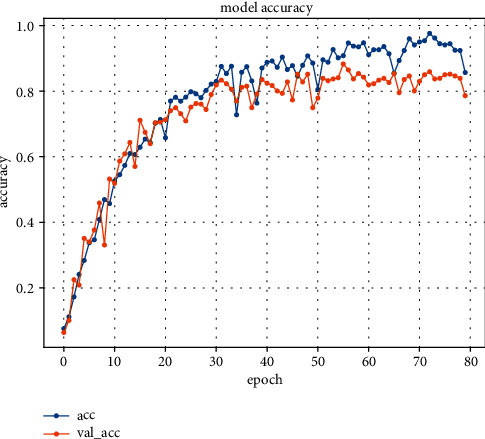
C3D model accuracy.

**Figure 11:**
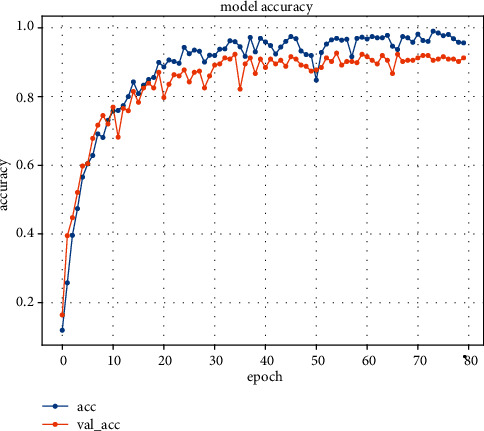
Proposed method accuracy.

Figures 10 and 11 show the change in the prediction accuracy of the C3D model and the model in this paper, respectively. The experiment carried out 80 rounds of training under 10 classifications and finally obtained the accuracy of the training set and the test set. It can be seen from the figure that the convergence speed of the C3D framework training is slow, and the curve rises erratically, resulting in oscillations. The overall trend of the method in this paper is rising and stable, the convergence speed is fast, and the final accuracy rate is about 9% higher than that of the traditional C3D model. The learning rate of these two methods is the same, so this may be because the C3D model cannot accurately capture the long-distance interdependence characteristics of complex actions, and the C3D model lacks normalization processing, resulting in singular values in the training process, which affect the speed and final accuracy of model learning.

In the comparative experiment, this study combined different network structures and verified the effectiveness of complex human motion classification on the UCF-101 dataset. The combination of lightweight 3D convolution and long short-term memory network is added in the experiment because, considering the reduced ability of the simplified C3D model to extract time series features, the LSTM network can well extract the context of video frames.

According to Table 2, the lightweight 3D convolutional network performs well on the 10-class classification problem, but the accuracy rate is significantly reduced with increasing the number of classifications. This may be because the depth of the lightweight 3D convolutional network is not enough. When faced with multi-classification tasks of complex motion, the lightweight model cannot extract richer features to distinguish different categories of videos. The performance of the classic C3D model is relatively stable, indicating that even if faced with more classification tasks, the C3D network architecture can still maintain a sure accuracy. Still, it needs to train more than 50 million parameters.

The combination of the Bi LSTM network and Lite-3DCNN has produced a specific result. Table 2 shows that the classification accuracy of the Lite-3dcnn combined with the LSTM framework is lower than that of the Lite-3DCNN model in the ten classification tasks. This is because when the number of classifications is small, the performance of the Lite-3dcnn model is good enough, and the advantages of the LSTM unit do not play a role. Still, the bidirectional LSTM unit extracts the information below the video frame to a certain extent, so it performs better than the C3D model. However, at 20 and 30 categories, the LSTM unit plays an advantage, making up for the simple structure of the Lite-3DCNN network. Even so, there is still no superior performance of the C3D model because the significant trainable parameters of the C3D model improve the ability of multi-classification tasks.

Experimental results show that the performance of the lightweight 3D convolutional architecture deteriorates with the increase in the number of classes. Although the method proposed in this paper also offers such a trend, the results are still better than the C3D framework on 30 classification tasks. The fundamental reason for this result is that the three-dimensional convolutional neural network can extract the spatio-temporal features of video data to a certain extent. The self-attention mechanism focuses on the global key features, increasing the receptive field with almost no increase in computational cost. Compared with the LSTM network, the self-attention mechanism and lightweight three-dimensional convolution network are better integrated, and more accurate prediction results are obtained.

### 4.3. Parameter Quantity Comparison

According to Table 3, the parameter amount of the method in this paper is only one-thirtieth of the C3D model. The accuracy is improved by about 4% in the 10–30 classification task. At the same time, in the case of adding a small number of parameters, the classification accuracy of the method in this paper is improved by about 9% on average compared with the lightweight 3D convolutional network, and the accuracy rate is still slightly higher than that of the C3D model when completing 30 classification tasks. This shows that the introduced self-attention method has played an important role, effectively making up for the deficiency of lightweight 3D convolution feature extraction capability. Although the combination of bidirectional LSTM and Lite-3DCNN produces some effect, the number of parameters is still about three times that of our model. Even under thirty categories, the trainable parameters of our model are only 1.884 M.

## 5. Conclusions

Complex human motion videos usually contain high-level semantic information and a large amount of redundant information. Although the classification framework based on the traditional three-dimensional convolution network can better complete the classification task, such a framework has many parameters. It requires a lot of time and computing power. This research introduces an efficient and lightweight human motion recognition framework, combining the lightweight C3D model and self-attention mechanism. The self-attention mechanism is used to capture critical global features. The receptive field is increased with only a few parameters, which makes up for the lightweight three-dimensional convolution network shortage. In the data processing stage, this study uses the average frame skipping sampling to reduce the data size as much as possible while retaining more complete features and uses the method of hot coding to enrich the data features and minimize interference. The experimental results show that, based on the ucf-101 dataset, the accuracy of the proposed method in the task of 10–30 classification is between 91.6% and 84.8%, which is about 5% and 10% higher than other models on average, and the parameter quantity is only one-thirtieth of that of the C3d model. However, the classification accuracy of the method proposed in this paper decreases slowly with the increase of categories, and no more classification experiments have been carried out in the study. In future research, we will consider combining the two-stream method and retraining in a more extensive dataset to improve the framework of this study further.

## Figures and Tables

**Table 1:** The network structure and parameters of this framework (20 class).

Layers	Output shape	Parameters
Input layer	32, 32, 20, 3	0
conv3d	32, 32, 20, 32	2624
activation	32, 32, 20, 32	655360
conv3d_1	32, 32, 20, 32	27680
activation_1	32, 32, 20, 32	0
max_pooling3d	10, 10, 6, 32	0
Dropout	10, 10, 6, 32	0
conv3d_2	10, 10, 6, 64	55360
activation_2	10, 10, 6, 64	0
conv3d_3	10, 10, 6, 64	110656
activation_3	10, 10, 6, 64	0
max_pooling3d_1	3, 3, 2, 64	0
dropout_1	3, 3, 2, 64	0
time_distributed (flatter)	3, 384	0
self__attention	3, 512	589824
Dense	3, 512	262656
batch_normalization	3, 512	2048
dropout_2	3, 512	0
global_average_pooling1d	512	0
dense_1	20	10260

**Table 2:** The validation accuracy of the proposed method for the complex human movement of the UCF-101 dataset.

Model	10 class (%)	20 class (%)	30 class (%)
C3D	82.2	84.7	83.5
Lite-3DCNN	85.3	80.2	70.6
Lite-3DCNN-LSTM	81.1	83.5	75.2
Lite-3DCNN-BiLSTM	84.5	85.3	79.5
Proposed method	**91.6**	**88.5**	**84.8**

**Table 3:** The trainable parameters (in millions) of the proposed method and other methods for the UCF-101 dataset.

Model type	10 class (M)	20 class (M)	30 class (M)
C3D	52.87	61.30	61.34
Lite-3DCNN	1.609	1.616	1.621
Lite-3DCNN–LSTM (512)	3.120	3.122	3.135
Lite-3DCNN–BiLSTM (512)	5.219	5.224	5.229
Proposed method	1.712	1.716	1.884

## Data Availability

The data used to support the findings of this study are available from the corresponding author upon request.
